# CD155/TIGIT signalling plays a vital role in the regulation of bone marrow mesenchymal stem cell–induced natural killer–cell exhaustion in multiple myeloma

**DOI:** 10.1002/ctm2.861

**Published:** 2022-07-20

**Authors:** Zhao‐Yun Liu, Ling Deng, Yue Jia, Hui Liu, Kai Ding, Wei Wang, Hongkai Zhang, Rong Fu

**Affiliations:** ^1^ Department of Hematology Tianjin Medical University General Hospital Tianjin P. R. China

Dear Editor,

T‐cell immunoreceptor with immunoglobulin and ITIM domain (TIGIT) is a potential immune checkpoint for natural killer (NK)‐cell exhaustion in multiple myeloma (MM), and its ligand, CD155 on bone marrow mesenchymal stem cells (BMSCs), is highly expressed in newly diagnosed MM (NDMM) but expressed at extremely low levels on myeloma cells. CD155/TIGIT signalling is involved in the regulation of BMSC‐induced NK‐cell exhaustion.

MM is an incurable disease, and most patients eventually relapse or develop drug‐resistant disease. BMSCs, an important cellular component of the bone marrow microenvironment, are critical for cell‐based immunotherapy because they modulate the functions of several immune cell types. BMSCs promote myeloma cell malignant proliferation in various ways. Meanwhile, myeloma cells can also reprogram BMSCs, which promotes the formation of a malignant regulatory loop based on BMSC–myeloma cell interactions.^1^, ^2^, ^3^, ^4^ NK cells are lymphocytes that kill tumour cells without prior sensitisation, because the expression of MHC class I is low on myeloma cells and they are easily recognised by NK cells at the early disease stage.^5^, ^6^ With disease progression, the expression of inhibitory immune checkpoints on NK cells is increased, leading to NK‐cell exhaustion and tumour cell immune escape. TIGIT is expressed on lymphocytes with an inhibitory role.^7^ Its immunoglobulin domain is similar to the amino terminal domain of CD226, both of which can bind their ligands CD155, CD112 and CD113.^8^ Competitive analysis showed that TIGIT has a higher affinity for CD155 than CD226, which can inhibit the interaction between CD155 and CD226 in a dose‐dependent manner.

Zavidij et al. demonstrated immune‐state changes in MM during early‐to‐late‐stage transition by single‐cell RNA sequencing (10× Genomics). They found that NK cells were increased during MM early stages and correlated with the expression of chemokine receptors.^9^ Their study revealed an overall change of immune cells in MM, but they did not focus on the inhibitory receptors on NK cells. We re‐analysed these sequencing results to explore the expression of TIGIT and CD96 on NK cells, which are both inhibitory receptors. TIGIT was highly expressed in monoclonal gammopathy of undetermined significance (*n* = 5), low‐risk smouldering myeloma (SMM) (*n* = 3), high‐risk SMM (*n* = 8) and NDMM (*n* = 7) compared to that in healthy controls (HCs) (*n* = 9), whereas CD96 expression was not significantly different (Figure 1).

**FIGURE 1:**
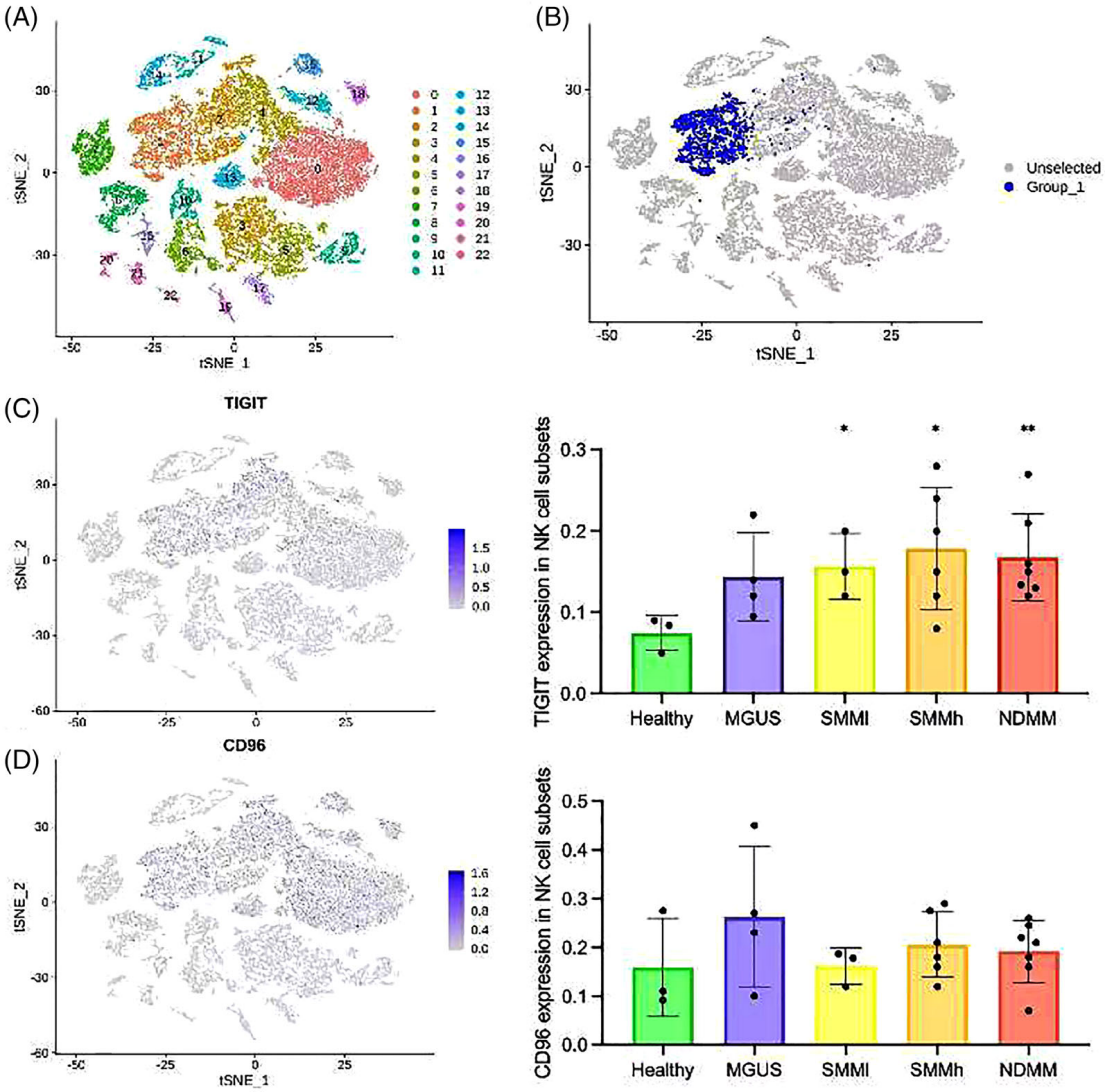
The co‐inhibitory receptor T‐cell immunoreceptor with immunoglobulin and ITIM domain (TIGIT) on natural killer (NK) cells is highly expressed in patients with multiple myeloma (MM), but the expression of CD96 is not significantly different. (A) *t*‐Distributed stochastic neighbour embedding (*t*‐SNE) representation of immune cells. Cluster 1 represents NK subsets identified in the CD3^−^CD19^−^CD14^−^CD56^+^ population after recalculation. (B) The blue dots represent the NK‐cell subsets provided in the previous paper, and our reanalysis coincided with this. (C) The distribution of TIGIT in *t*‐SNE (left) and its expression (right) in NK‐cell subsets, using a two tailed *t*‐test. TIGIT expression at each stage (monoclonal gammopathy of undetermined significance [MGUS], low‐risk smoldering myeloma [SMMl], high‐risk SMM [SMMh], newly diagnosed multiple myeloma [NDMM]) of disease progression was compared with that in healthy control samples, and *p*‐values were .093, .025, .021 and .004, respectively. (D) The distribution of CD96 in *t*‐SNE (left) and its expression (right) in NK‐cell subsets, using a two‐tailed *t*‐test. *p*‐Values for the CD96 expression levels at stages (MGUS, SMMl, SMMh, NDMM) of disease progression were calculated and compared with those in healthy controls, and *p*‐values were .153, .475, .102 and .139, respectively; therefore, no statistical significance was observed. *p <  .05, **p <  .01. Patients with SMM were stratified by the risk of progression into SMMl and SMMh, based on the criteria established by the Mayo Clinic.

Next, we used clinical samples to verify the significant high expression of TIGIT in MM (Table S1). TIGIT expression was higher in NDMM (61.46% ± 12.51%) than in the CR (complete response) group (19.52% ± 13.36%) and in HCs (13.80% ± 5.075%) (both *p *< .01). Expression of the active receptor CD226 was lower in the NDMM group (50.66% ± 13.59%) than in the CR (84.99% ± 8.390%) and HC (86.43% ± 13.26%) groups (both *p *< .01; Figure 2B,E). Activated receptors (NKG2D and CD107a) and functional biomarkers (IFN‐γ and perforin) on NK cells were decreased significantly in NDMM patients (Figure 2C,E). On NK cells, changes in levels of CD226, TIGIT, NKG2D, CD107a, the function marker perforin and IFN‐γ in bone marrow were consistent with those in peripheral blood (Tables S2 and S3). The expression of activated and functional molecules on TIGIT^+^NK cells was lower than that on TIGIT^−^NK cells, whereas those on CD226^+^NK cells were elevated (Figure 2D), indicating that TIGIT expression was significantly increased and correlated with NK‐cell modulation.

**FIGURE 2:**
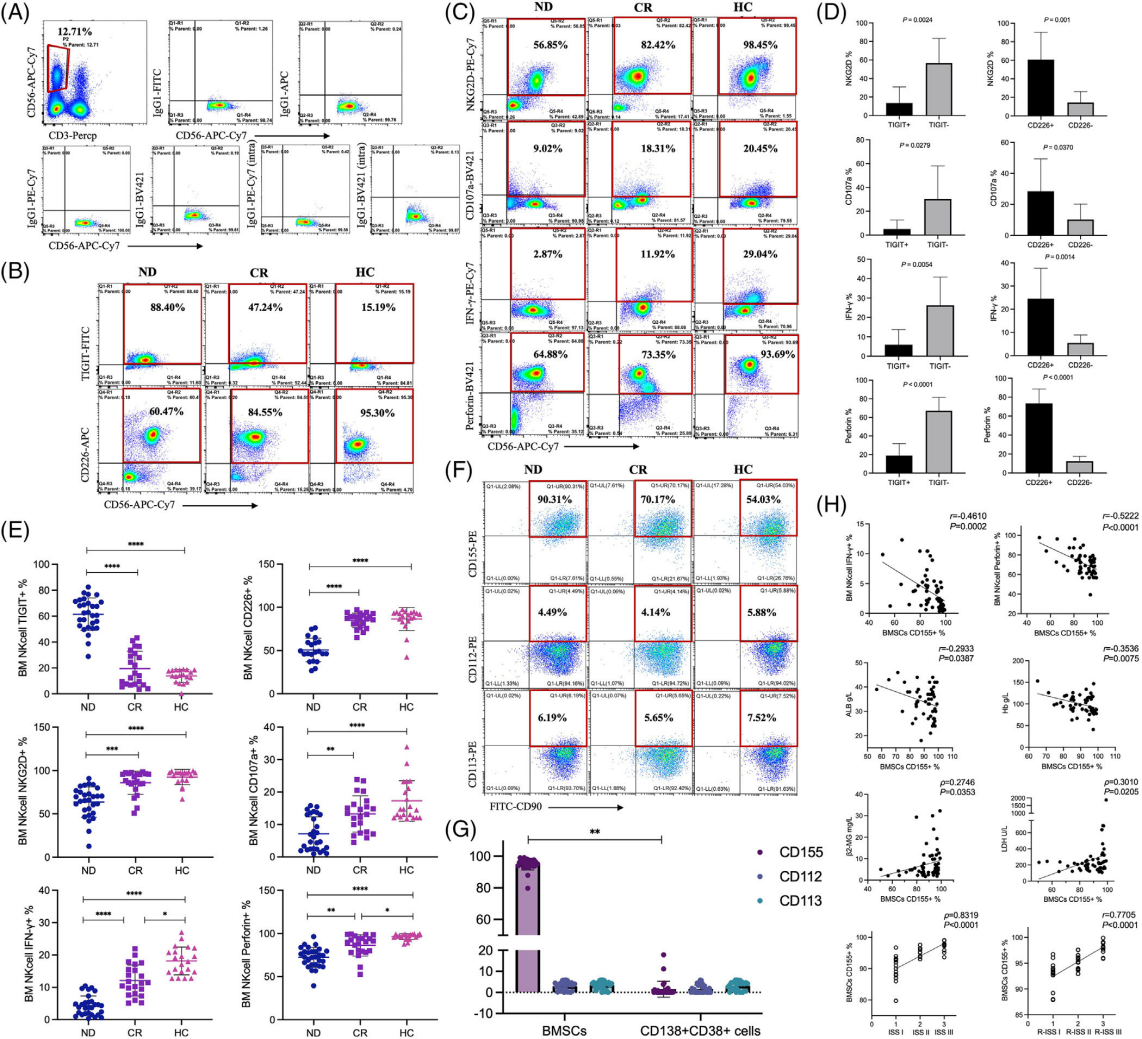
In multiple myeloma (MM) patients, the expression of T‐cell immunoreceptor with immunoglobulin and ITIM domain (TIGIT) is significantly increased on natural killer (NK) cells, and its ligand CD155, but not CD112 and CD113, is highly expressed on bone marrow mesenchymal stem cells (BMSCs). (A) NK cells were represented as CD3^−^CD56^+^ by performing flow cytometry. Fluorescein‐labelled IgG1 was used as an isotype control. (B) The expression of TIGIT was significantly increased on NK cells from newly diagnosed multiple myeloma (NDMM) patients compared to that in healthy control (HC) samples, whereas CD226 expression was decreased. (C) The expression of NK‐cell surface‐activated molecules NKG2D and CD107a and NK‐cell functional molecules IFN‐γ and perforin were decreased in NDMM patients. These levels were increased gradually in patients with complete response (CR) patients and HCs. These results were detected using spectral flow cytometry. (D) Frequency of cells expressing NKG2D, CD107a, IFN‐γ, and perforin among TIGIT^+^NK cells or TIGIT^−^NK cells. The expression of activated and functional molecules in TIGIT^+^NK cells was lower than that on TIGIT^−^NK cells, whereas those on CD226^+^NK cells were elevated. (E) The dot plots are the statistics of (B) and (C) and include patients with NDMM (*n* = 52), CR patients (*n* = 47) and HCs (*n* = 40). (F) The expression of CD155 was greatly upregulated on BMSCs of MM patients, but CD112 and CD113 were expressed at very low levels. In addition, the expression of CD155 on BMSCs of NDMM patients was higher than that in cells from CR patients and HCs. (G) Ligand expression levels of CD155, CD112 and CD113 on BMSCs were detected using flow cytometry. (H) The correlation between CD155 expression on BMSCs in NDMM patients and NK‐cell functional molecules, IFN‐γ and perforin and clinical characteristics, including β2‐MG, LDH, ALB, Hb, ISS stage and R‐ISS stage. *p <  .05, **p <  .01, ***p <  .001 and ****p <  .0001

As the interaction between the bone marrow environment and NK cells has been reported,^10^ we speculated that the expression of CD155, CD112 and CD113, ligands of TIGIT and CD226, might be expressed on BMSCs and even myeloma cells. Interestingly, the expression of CD155 was greatly upregulated on BMSCs from NDMM patients (95.07% ± 3.644%), but CD112 and CD113 were expressed at very low levels (Figure 2F). However, CD155, CD112 and CD113 levels were very low in myeloma cells (Figures 2G and S2C). Furthermore, levels of the NK‐cell functional molecules perforin and IFN‐γ were negatively associated with CD155 expression on BMSCs. The expression of CD155 was closely associated with MM clinical characteristics (Figure 2H). Considering that the TIGIT ligand CD155 was highly expressed on BMSCs, we speculated that its immunosuppressive functions in NK cells occur via the interaction with CD155 on BMSCs.

Next, we revealed the mechanism through which BMSCs regulate NK cells via CD155/TIGIT using in vitro co‐culture (Figure 3A). After NK cells were amplified with IL‐2, expression of the activated markers NKG2D, NKp44, CD69 and NKp30 on NK cells were increased, but these levels were decreased after NK‐cell co‐culture with BMSCs for 6 days (Figure S3B). In co‐culture systems, TIGIT mAb and TIGIT^+^CD226 mAb groups showed restored expression of these markers on NK cells and IFN‐γ secretion was increased in NK cells (Figure 3B left, C). To verify the changes in NK‐cell function, we added the U266 cell line to co‐culture systems; apoptosis of U266 gradually increased after adding TIGIT mAbs (Figure 3B right). We further detected related protein changes in the CD155/TIGIT signalling pathway; here, SHIP‐1 expression decreased and Erk phosphorylation increased with TIGIT blockade (Figure 3D). As TIGIT binds to CD155 with higher affinity and out‐competes CD226 for CD155 binding, we purified the agonist CD226 mAb (WO 2020/023312 A1) to activate CD226 while suppressing TIGIT (Figure S3E). A blocking TIGIT mAb and agonist CD226 mAb were added to BMSC and NK‐cell co‐culture systems, and we found that the activation of NK cells increased with TIGIT combined with the agonist CD226 mAb and IFN‐γ secretion, and U266 cell apoptosis was elevated (Figure S3C,D,F).

**FIGURE 3:**
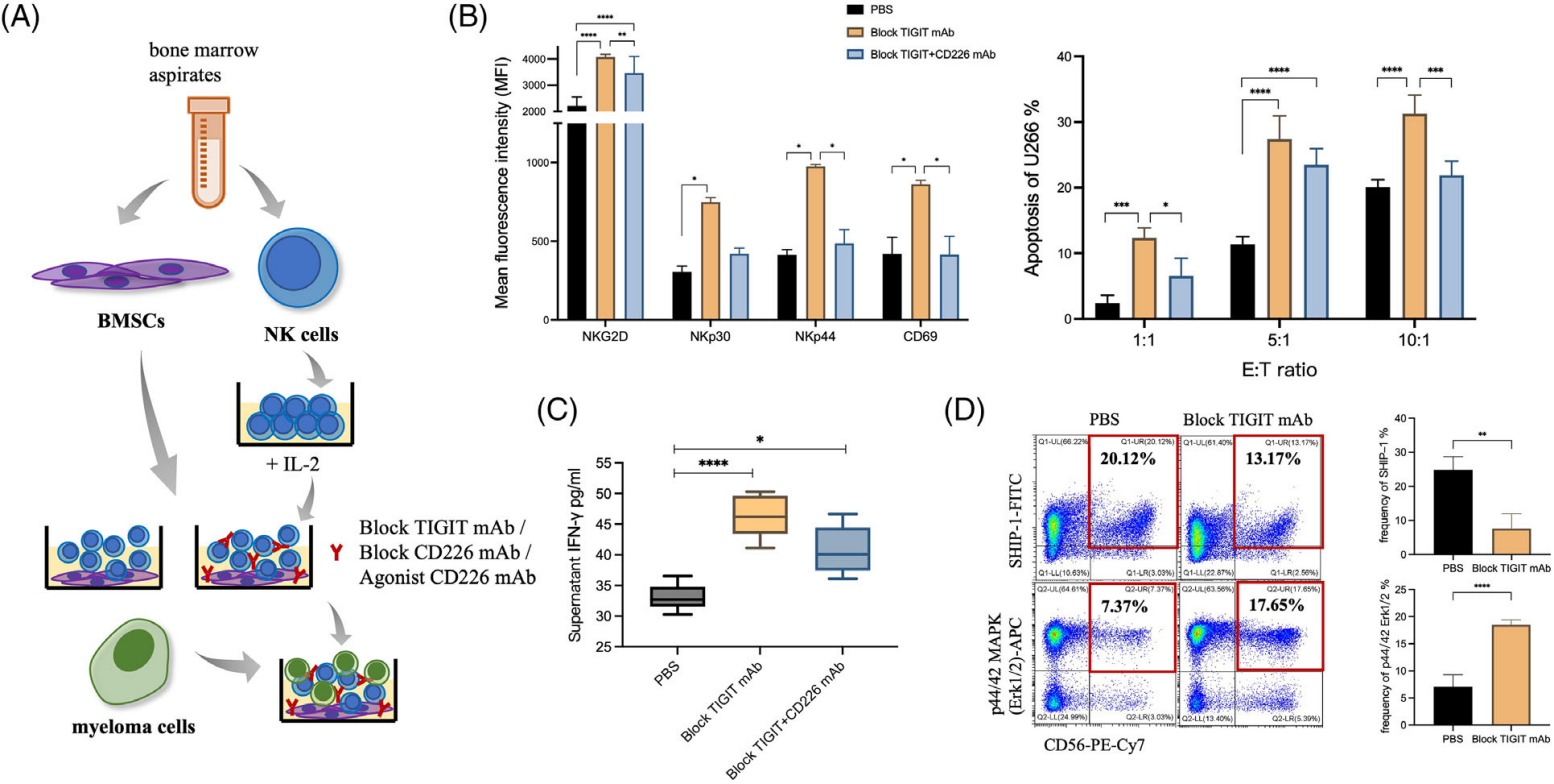
Blockade of T‐cell immunoreceptor with immunoglobulin and ITIM domain (TIGIT) might restore the expression of activated markers and killing function of natural killer (NK) cells. (A) Experimental process of bone marrow mesenchymal stem cell (BMSC) and NK‐cell co‐culture. (B) The expression of markers of activated NK cells, NKG2D, NKp30, NKp44 and CD69 was detected using flow cytometry after blocking TIGIT and CD226 mAbs were added. TIGIT mAbs and TIGIT^+^CD226 mAbs groups showed restored expression of these activated markers on NK cells. The results are presented as mean fluorescence intensity (MFI) (left). We added the U266 cell line into the co‐culture systems, and the apoptosis of U266 cells gradually increased after adding TIGIT mAbs (right). (C) IFN‐γ secretion was detected using flow cytometry and was increased in NK cells after the blockade of TIGIT. (D) Expression levels of SHIP‐1 and pErk in the TIGIT/CD155 signalling pathway were detected using flow cytometry. Moreover, SHIP‐1 expression decreased and Erk phosphorylation increased with the blockade of TIGIT. *p <  .05, **p <  .01, ***p <  .001 and ****p <  .0001

In summary, we demonstrated that TIGIT was upregulated, but that CD226 was downregulated, on NK cells from NDMM patients. Regarding the TIGIT ligand, CD155, but not CD112 and CD113, was highly expressed on BMSCs but was expressed at low levels on myeloma cells. In vitro co‐culture results demonstrated that CD115/TIGIT signalling plays a vital role SI in the interaction between BMSCs and NK cells. Blocking TIGIT could greatly restore NK‐cell exhaustion, providing a potential avenue for antitumour immunotherapy for MM (Figure 4).

**FIGURE 4:**
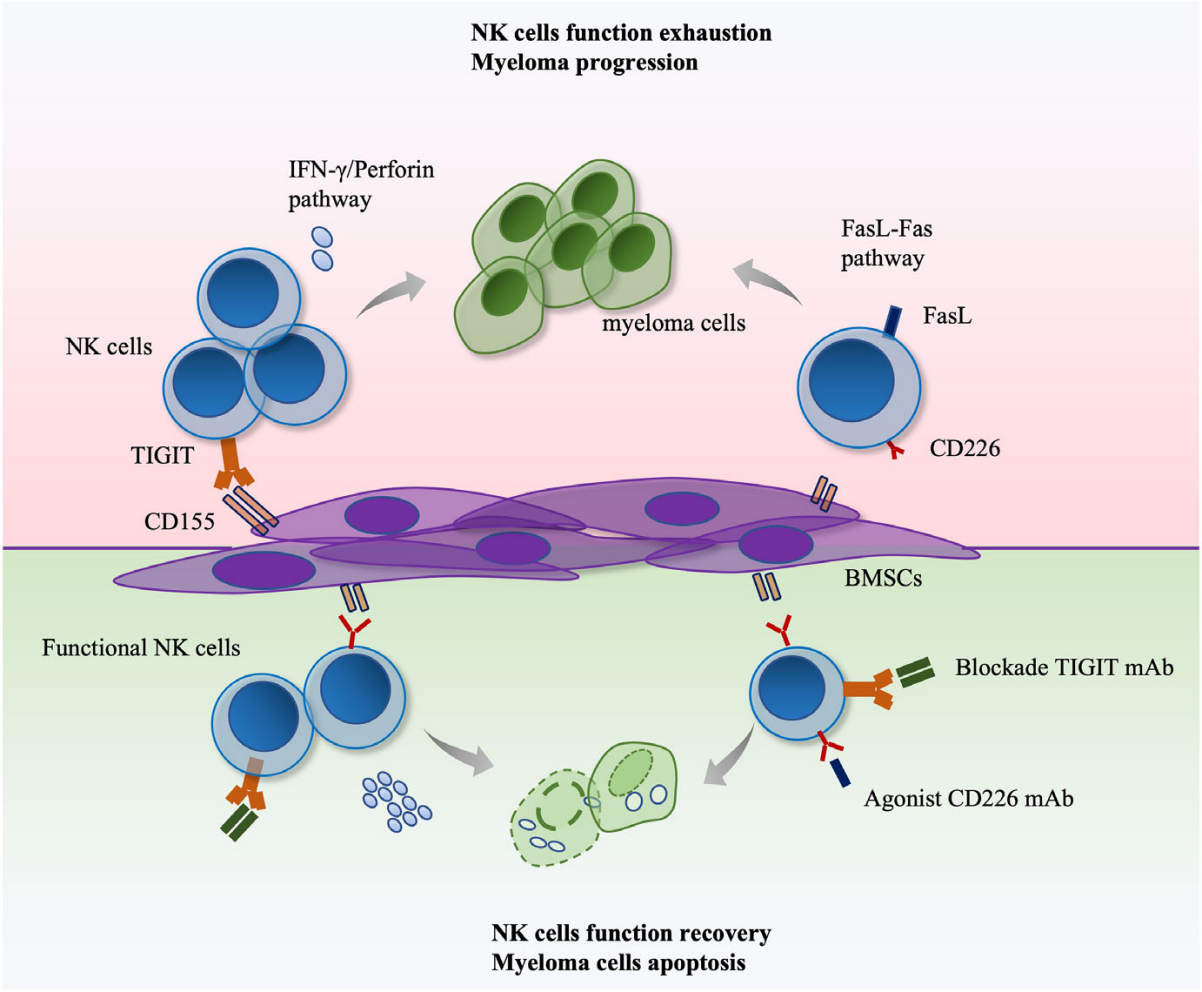
T‐cell immunoreceptor with immunoglobulin and ITIM domain (TIGIT) blockade might restore natural killer (NK)‐cell exhaustion by bone marrow mesenchymal stem cells (BMSCs) and inhibit myeloma progression.

## CONFLICT OF INTEREST

All authors declare no conflict of interest.

## Supporting information

Figure S1 Natural killer (NK)‐cell individual markers in the immune landscape of multiple myeloma (MM).
Figure S2 Identification of bone marrow mesenchymal stem cells (BMSCs) and the ligand expression of CD155, CD112, and CD113 are extremely low on myeloma cells.
Figure S3 Blockade of TIGIT and activating CD266 might better restore natural killer (NK)‐cell function.

Supporting Information

Supporting Information

Supporting Information

Supporting Information

Supporting Information

Supporting Information
