# Supplementary material for: CD155/TIGIT signalling plays a vital role in the regulation of bone marrow mesenchymal stem cell–induced natural killer–cell exhaustion in multiple myeloma
Source: Clin Transl Med. 2022 Jul 20;12(7):e861. doi: 10.1002/ctm2.861 (PMC9299950; doi:10.1002/ctm2.861)
Supplement: Supplementary file 1 — Figure S1 Natural killer (NK)‐cell individual markers in the immune landscape of multiple myeloma (MM). Figure S2 Identification of bone marrow mesenchymal stem cells (BMSCs) and the ligand expression of CD155, CD112, and CD113 are extremely low on myeloma cells. Figure S3 Blockade of TIGIT and activating CD266 might better restore natural killer (NK)‐cell function. [file CTM2-12-e861-s003.docx]

1. Supplementary
2. Figure S1.


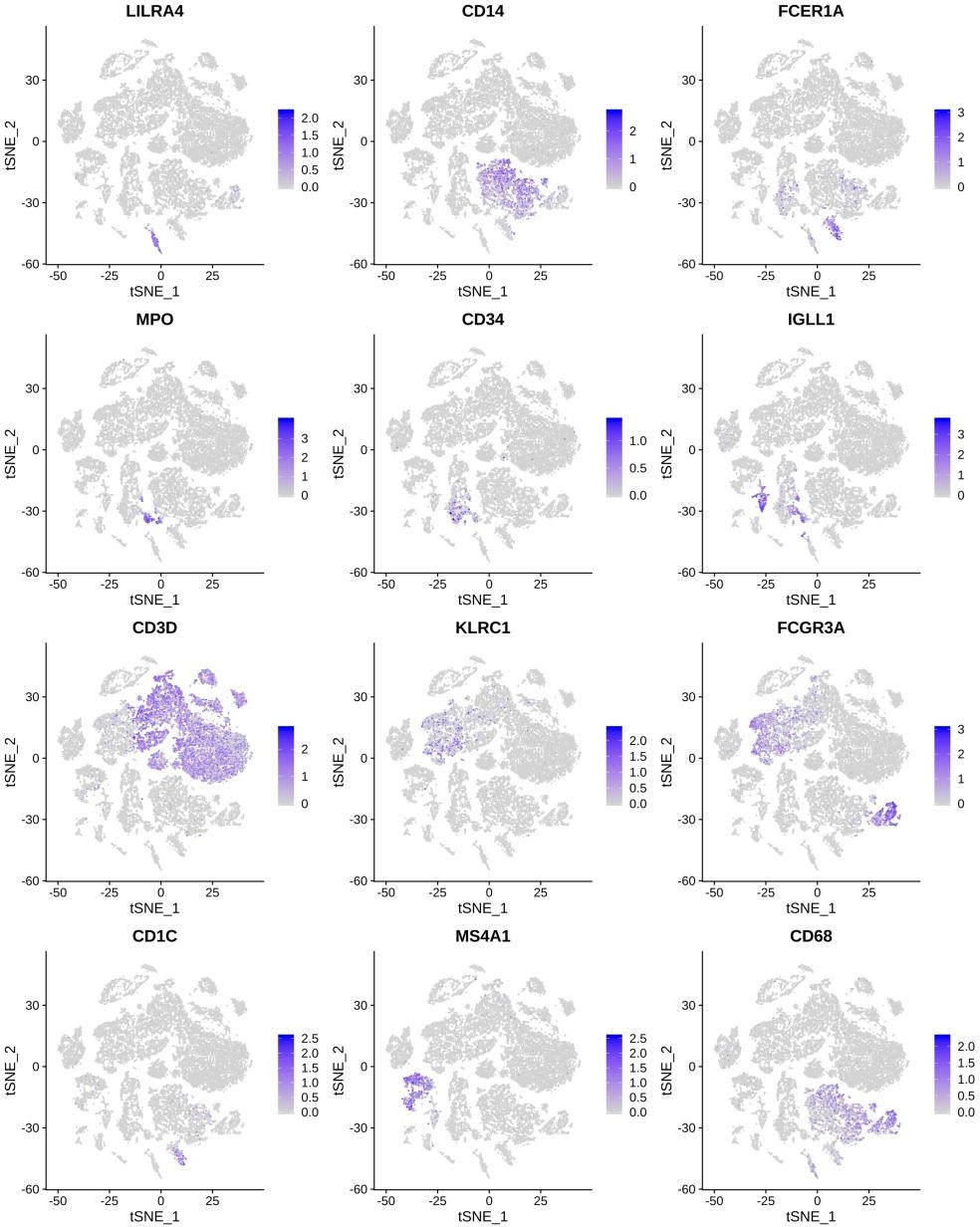
A

B NK cells in Cluster 1

155

2009

91

NK cells in Paper

242

1. Figure S1. Natural killer (NK) cell individual markers in the immune landscape of multiple
2. myeloma (MM).
3. A. Distribution of NK cell individual markers in tSNE. B. The overlap of our recalculation and
4. original results from the above paper. After recalculation, the distribution of NK cell subsets and
5. their individual markers were consistent with those provided in the above paper, and the accuracy
6. and coverage reached more than 90%.
7. 249
8. 250
9. 251
10. 252
11. 253
12. 254
13. 255
14. 256

Figure S2.

# A


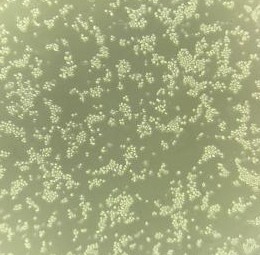

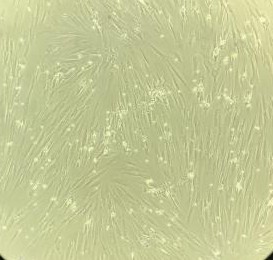

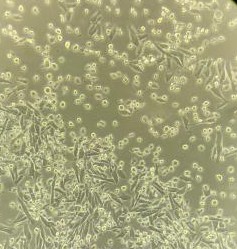

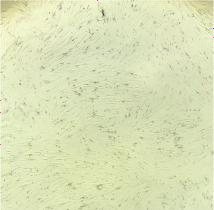


a

b

40×

200×

c

d

40×

100×

C


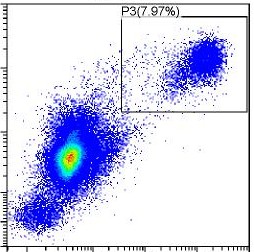


**7.97%**

# B


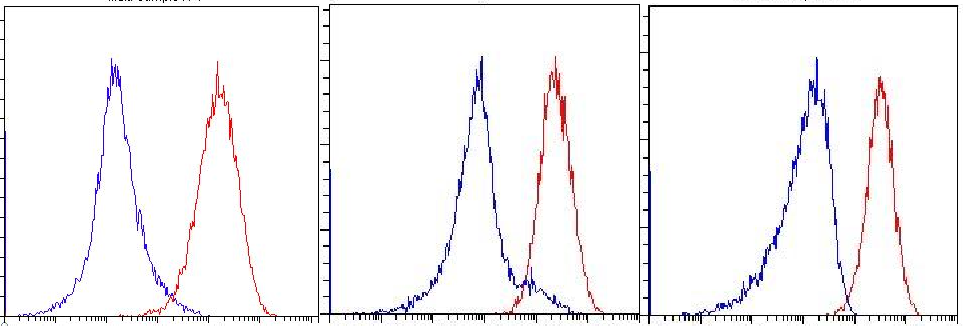

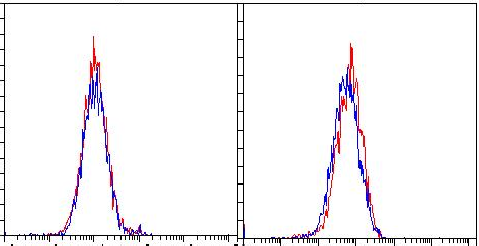
CD105-APC CD90-FITC CD73-BV510

IgG1 isotope

BMSCs identified marker

Count

CD45-APC-Cy7 CD34-Percp

1. 257


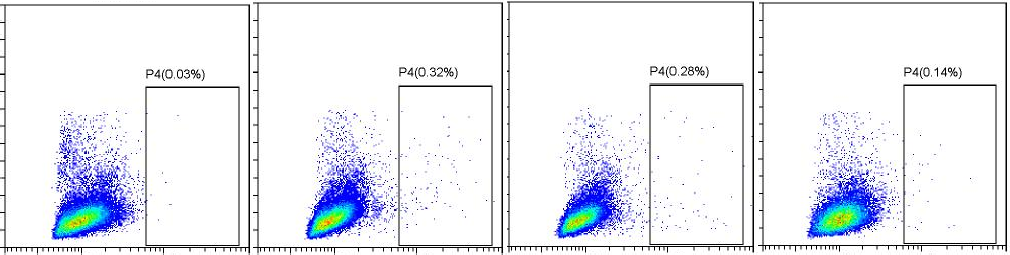


**0.32%**

**0.28%**

**0.14%**

CD38-FITC

SSC

CD138-BV510 IgG1-PE

CD155-PE CD112-PE CD113-PE

1. Figure S2. Identification of bone marrow mesenchymal stem cells (BMSCs) and the ligand
2. expression of CD155, CD112, and CD113 are extremely low on myeloma cells.
3. A. Primary BMSCs were cultured in vitro. A-a represents bone marrow mononuclear cell adherent
4. culture for 3 days, and a small number of cell colonies was observed under the microscope. A-b
5. indicates adherent culture for 7 days, and the number of adherent cells with irregular morphology
6. was observed. A-c and A-d indicate BMSCs after passage, showing a uniform and fibrous cell
7. morphology. B. BMSCs were defined as CD105^+^CD90^+^CD73^+^CD45^−^CD30^−^ cells using flow
8. cytometry, and the purity reached 90%. C. Ligand expression levels of CD155, CD112, and
9. CD113 on myeloma cells was determined using flow cytometry; the expression levels were
10. extremely low. Myeloma cells were represented by CD138^+^CD38^+^ cells and fluorescein-labelled
11. IgG1 as an isotype control.
12. 269
13. 270
14. 271
15. 272
16. 273
17. 274
18. 275
19. 276
20. 277
21. Figure S3.


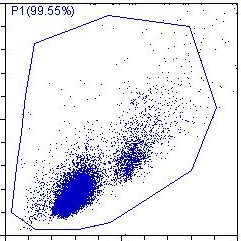

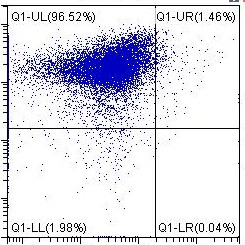
A B **3000**

NK day 0

NK day 6

Co-culture da

**0**

Mean fluorescence intensity (MFI)

**2000**

**1000**

**600**

CD56-PE

**400**

SSC

PBS Block TIGIT mAb Agonist CD226 mAb

y 6


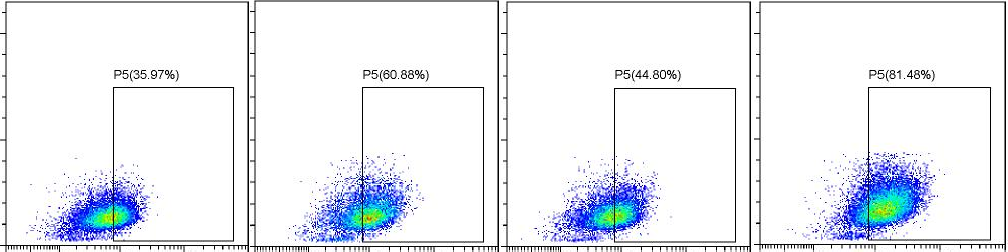


**35.97%**

**60.88%**

**44.80%**

**81.48%**

F

Block TIGIT + Agonist CD226 mAb

C

100

FSC

CD3-APC

**200**

NKG2D

NKp30

NKp44

CD69

NKG2D-PE-Cy7

80 80


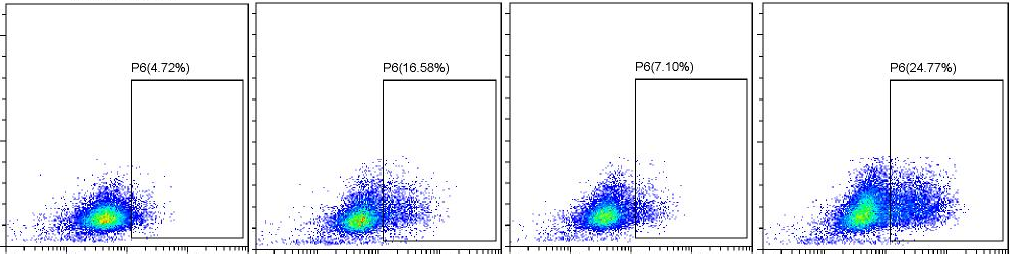


**4.72%**

**16.58%**

**7.10%**

**24.77%**

60 60

frequency on NK cells %

Apoptosis of U266 %

40

20

0

NKG2D

D

Supernatant IFN-γ pg/ml

500

400

300

200

100

0

NKp30

NKp40

CD69

40

20

0

1:1

E

5:1

10:1

180kDa

80kDa 60kDa

45kDa 35kDa

25kDa

PBS

Block TIGIT mAb Agonist CD226 mAb

Block TIGIT + Agonist CD226 mAb

Agonist CD226 mAb


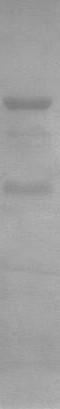

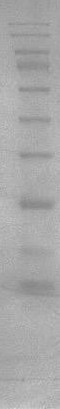


H

L

NKp30-BV510


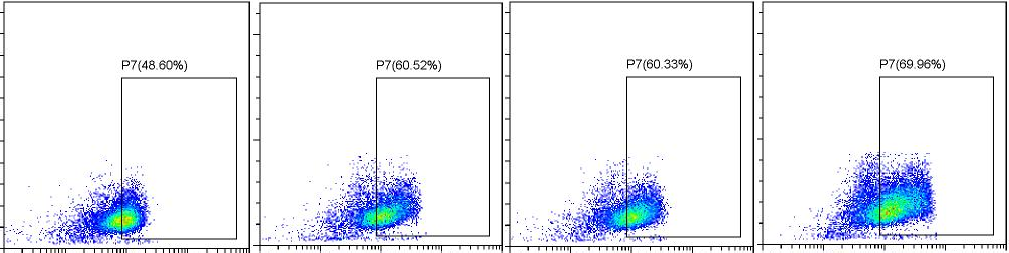


**48.60%**

**60.52%**

**60.33%**

**69.96%**

NKp44-PE


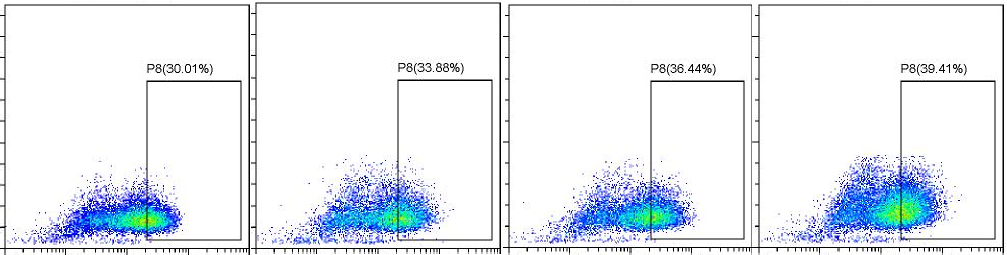


**30.01%**

**33.88%**

**36.44%**

**39.41%**

CD69-FITC

SSC

279

1. Figure S3. Blockade of TIGIT and activating CD266 might better restore natural killer (NK) cell
2. function.
3. A. Isolated NK cells from bone marrow mononuclear cells from newly diagnosed multiple
4. myeloma (NDMM) patients using MACS. Subsequent experiments were performed when NK cell
5. purity reached 95% (detected using flow cytometry). B. Expression of NKG2D, NKp30, NKp44,
6. and CD69, detected using flow cytometry, on NK cells after IL-2 activation and co-culture with
7. bone marrow mesenchymal stem cells (BMSCs). After NK cells were amplified with IL-2, the
8. expression of the above activated markers was increased but decreased after NK cells were
9. co-cultured with BMSCs for 6 days. C. Expression of NKG2D, NKp30, NKp44, and CD69,
10. detected using flow cytometry, on NK cells after blocking TIGIT mAbs and agonist CD226 mAbs
11. were added (left), and apoptosis of U266 cells in NK cells and BMSC co-culture systems, detected
12. using flow cytometry (right). The expression of markers of activate NK cells was increased, and
13. U266 apoptosis were elevated after treatment with blocking TIGIT mAbs combined with agonist
14. CD226 mAbs. D. The concentration of IFN-γ was detected by flow cytometry using the co-culture
15. supernatants of BMSCs and NK cells, and IFN-γ secretion was elevated after blocking TIGIT
16. mAbs and CD226 mAbs were added. E. The heavy and light chain expression plasmids for the
17. agonist CD226 mAb were co-transfected into HEK293T cells using Lipofectamine, and the
18. agonist CD226 mAbs purified from the culture supernatant of transfected HEK293T cells were
19. analysed using 10% SDS-PAGE. F. The expression of NKG2D, NKp30, NKp44, and CD69,
20. detected using flow cytometry, in NK cell and BMSC co-culture systems was detected using flow

300 cytometry. **P* < 0.05, ***P* < 0.01, ****P* < 0.001, and *****P* < 0.0001.

301


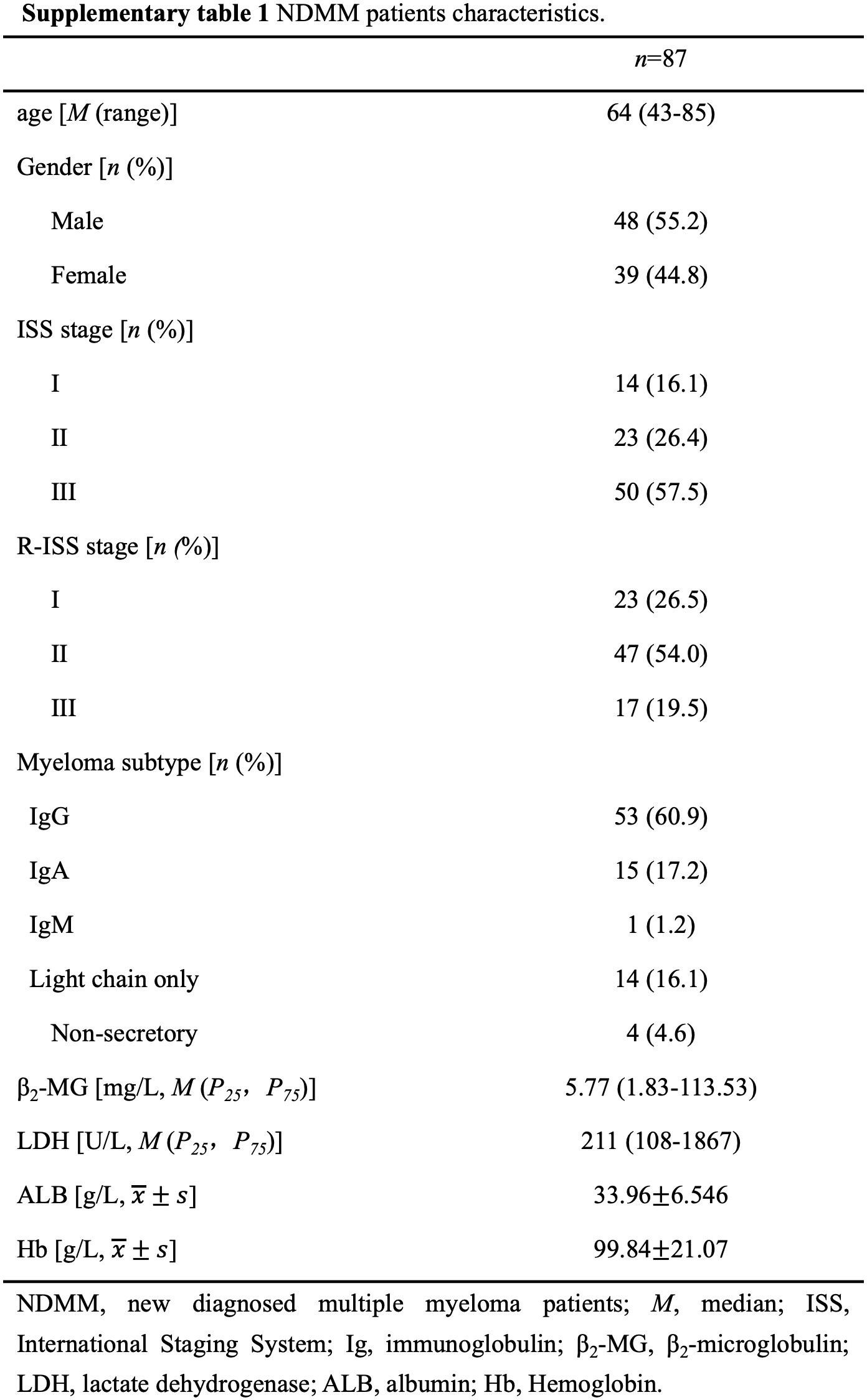
302


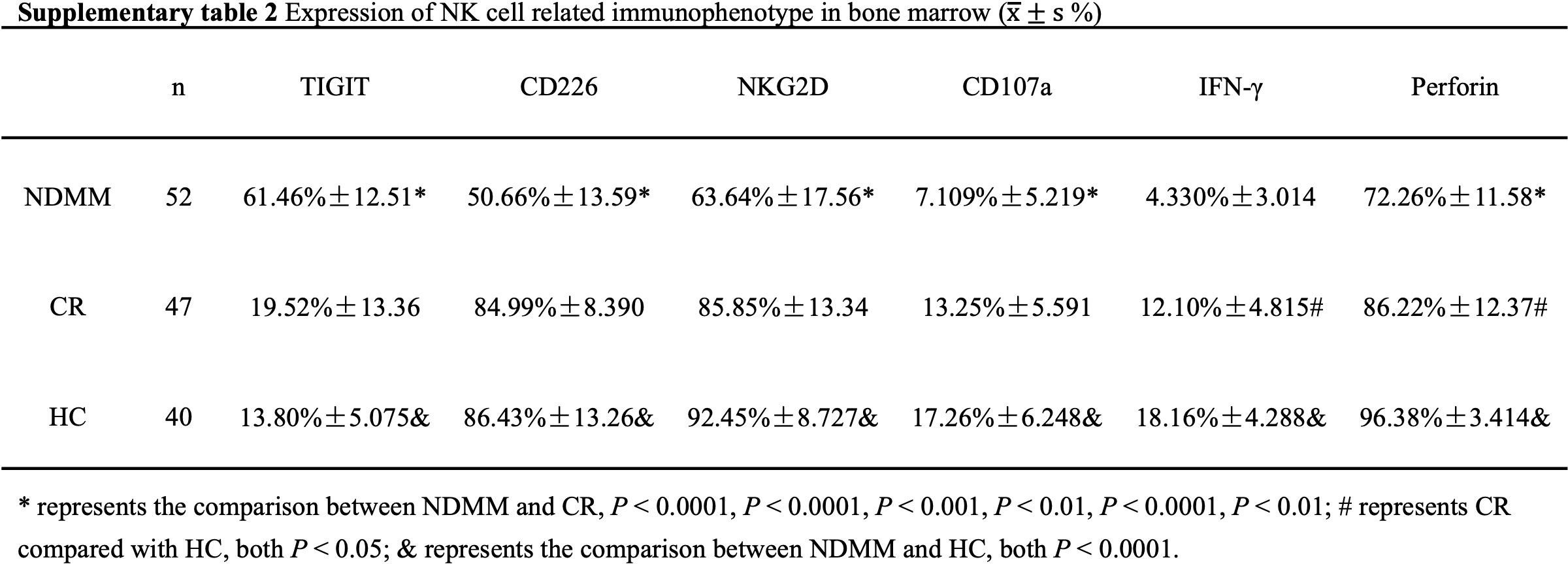
303


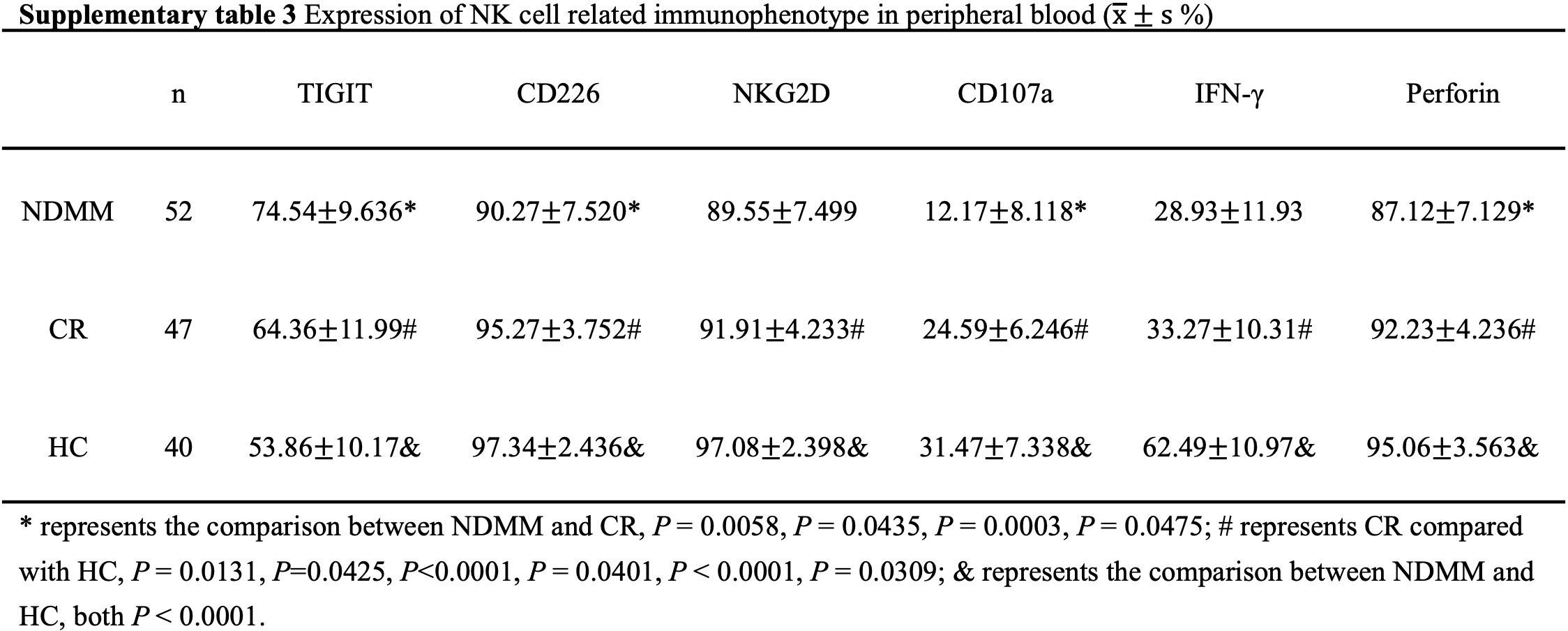
304

305

306

1. Methods
2. Patients
3. In total, 123 MM patients admitted to the Haematology Department of Tianjin Medical
4. University General Hospital between July 2018 and December 2019 were included in this study.
5. According to the 2014 International Myeloma Working Group (IMWG) diagnostic criteria and
6. 2016 IMWG response criteria, MM patients included 87 NDMM patients and 64 CR patients
7. (including 28 NDMM patients after treatment and 36 patients with a sustained complete response).
8. Patients with NDMM were staged according to the International Staging System (ISS) and the
9. Revised International Staging System (R-ISS). The HC group consisted of 54 healthy volunteers
10. were matched for age and sex. The study protocol was approved by the Ethics Committee of
11. Tianjin Medical University General Hospital in accordance with the Declaration of Helsinki, and
12. all subjects provided signed informed consent. The basic information regarding NDMM patients is
13. provided in Supplementary Table 1.
14. 320
15. BMSCs and NK cells
16. Bone marrow was harvested from the iliac crest of patients with MM and healthy volunteers.
17. Bone marrow mononuclear cells (BMMNCs) were isolated from bone marrow aspirates by
18. density-gradient centrifugation (Solarbio). BMMNCs were resuspended in T25 flasks and the cell
19. number was adjusted to 10^6^ cells/mL in Dulbecco’s modified Eagle medium (DMEM)/F-12
20. complete medium containing 15% foetal bovine serum (FBS), 100 U/mL penicillin, and 100
21. μg/mL streptomycin (all obtained from Life Technologies). After incubation at 37 °C in a 5% CO2
22. humidified atmosphere for 72 h, the non-adherent cells were removed, and the medium was
23. changed twice per week. When sub-confluency reached 80%–90%, the cells were digested with
24. 0.25% trypsin (Life Technologies) and expanded. BMSCs were defined according to the
25. International Society for Cellular Therapy (ISCT) criteria.
26. Primary NK cells were purified from BMMNCs using an NK Cell Isolation Kit based on
27. MACS system technology (Miltenyi Biotec GmbH, Bergisch, Germany) following the
28. manufacturer’s instructions. As assessed by flow cytometry, the purity of the isolated cells was
29. always greater than 95%. For NK cell activation, IL-2 (10 ng/mL; R&D Systems) was used.
30. 336
31. Co-culture of BMSCs and NK cells
32. BMSCs from passage two (P2) were plated at 5 × 10^4^ cells/cm^2^ in a flat-bottomed 24-well
33. plate. Following overnight adherence, activated NK cells were added to BMSCs (cell ratio of 5:1),
34. and the culture was maintained for 5 days in RPMI-1640 medium supplemented with 10% FBS
35. (Life Technologies). NK cells were pre-activated with IL-2 and then added to the BMSCs. A
36. functional grade TIGIT monoclonal antibody was used to block TIGIT (Thermo Fisher Scientific).
37. 343
38. Flow cytometry
39. The expression of TIGIT, CD226, activated NK cell markers, and functional molecules in
40. NDMM patients, CR patients, and HCs were detected using spectral flow cytometry (Cytek
41. NL-CLC) and analysed using spectro Flow software V1.0.1. Flow cytometry was conducted in
42. co-culture experiments using CytoFLEX and analysed using Flowjo V10.
43. 349
44. Western blotting analysis
45. Supernatants from transfected cells were subjected to 4%–12% Bis-Tris sodium dodecyl
46. sulphate-polyacrylamide gel electrophoresis (SDS-PAGE), and the protein bands in the gel were
47. transferred to polyvinylidene difluoride (PVDF) membranes (Invitrogen). The membranes were
48. incubated with HRP-conjugated goat anti-mouse IgG (H+L) (Pierce), and immunoreactive bands
49. were visualised using a horseradish peroxidase (HRP) chromogenic substrate (Invitrogen).
50. 356
51. Statistical analysis
52. The measurement data in line with the normal distribution are described as the mean ± standard
53. deviation. An independent sample t-test or paired sample t-test was used to analyse differences
54. between two groups. One-way or two-way ANOVA was used to analyse differences between three
55. or more groups, and the correlation between data was described by the Pearson correlation
56. coefficient (R). When the data displayed a skewed distribution, the median (interquartile spacing)
57. [M (P25, P75)] was used, and non-parametric tests were used to compare whether the difference
58. was significant. The Spearman rank correlation coefficient (ρ) was used to describe the correlation
59. between data points. Statistical significance was set at P < 0.05. GraphPad Prism 8 was used to
60. analyse data and generate statistical graphs.
