# Supplementary figures and images for: CD155/TIGIT signalling plays a vital role in the regulation of bone marrow mesenchymal stem cell–induced natural killer–cell exhaustion in multiple myeloma
Source: Clin Transl Med. 2022 Jul 20;12(7):e861. doi: 10.1002/ctm2.861 (PMC9299950; doi:10.1002/ctm2.861)

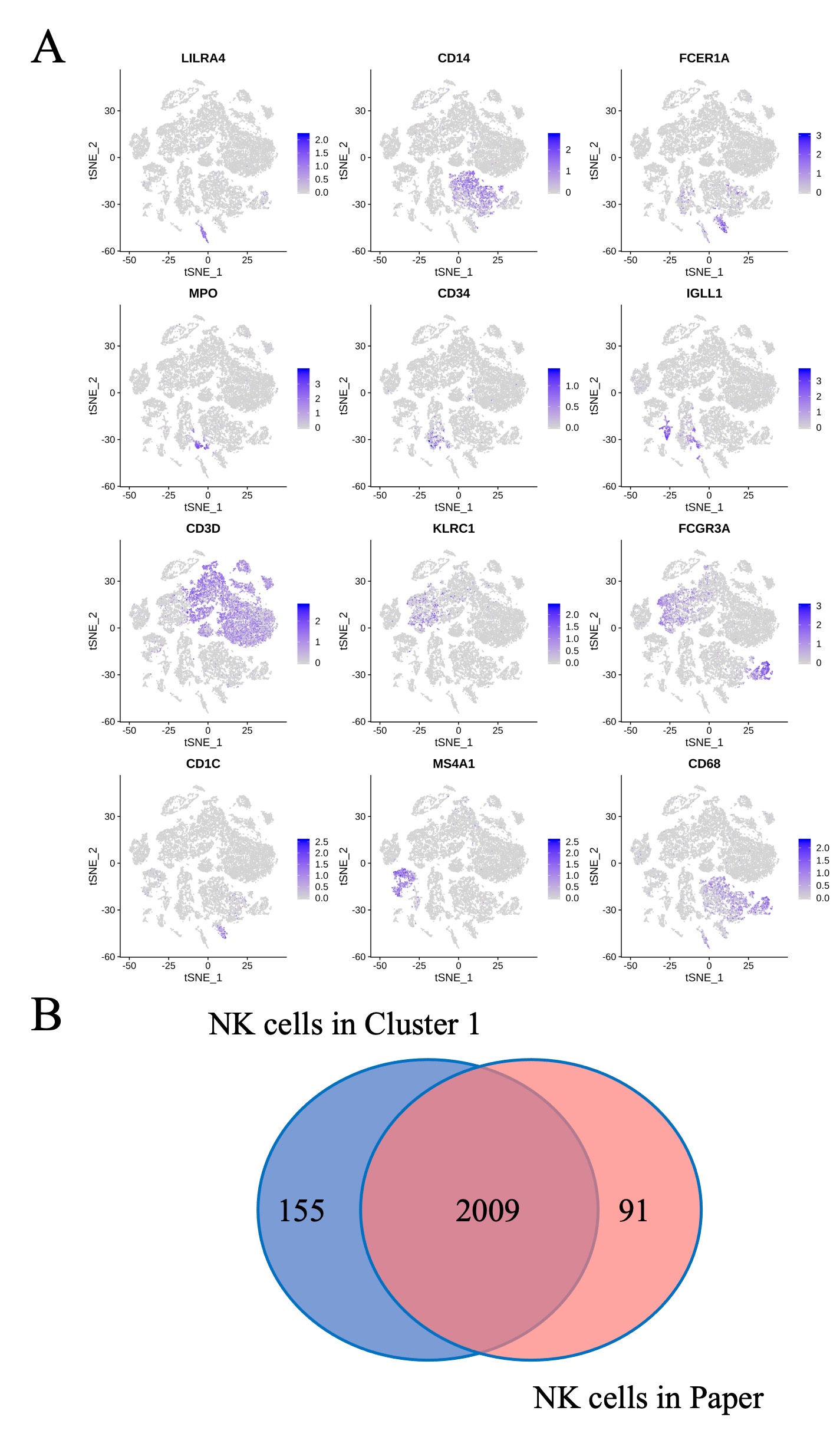

Supplement: Supplementary file 2 — Supporting Information [file CTM2-12-e861-s004.jpg]

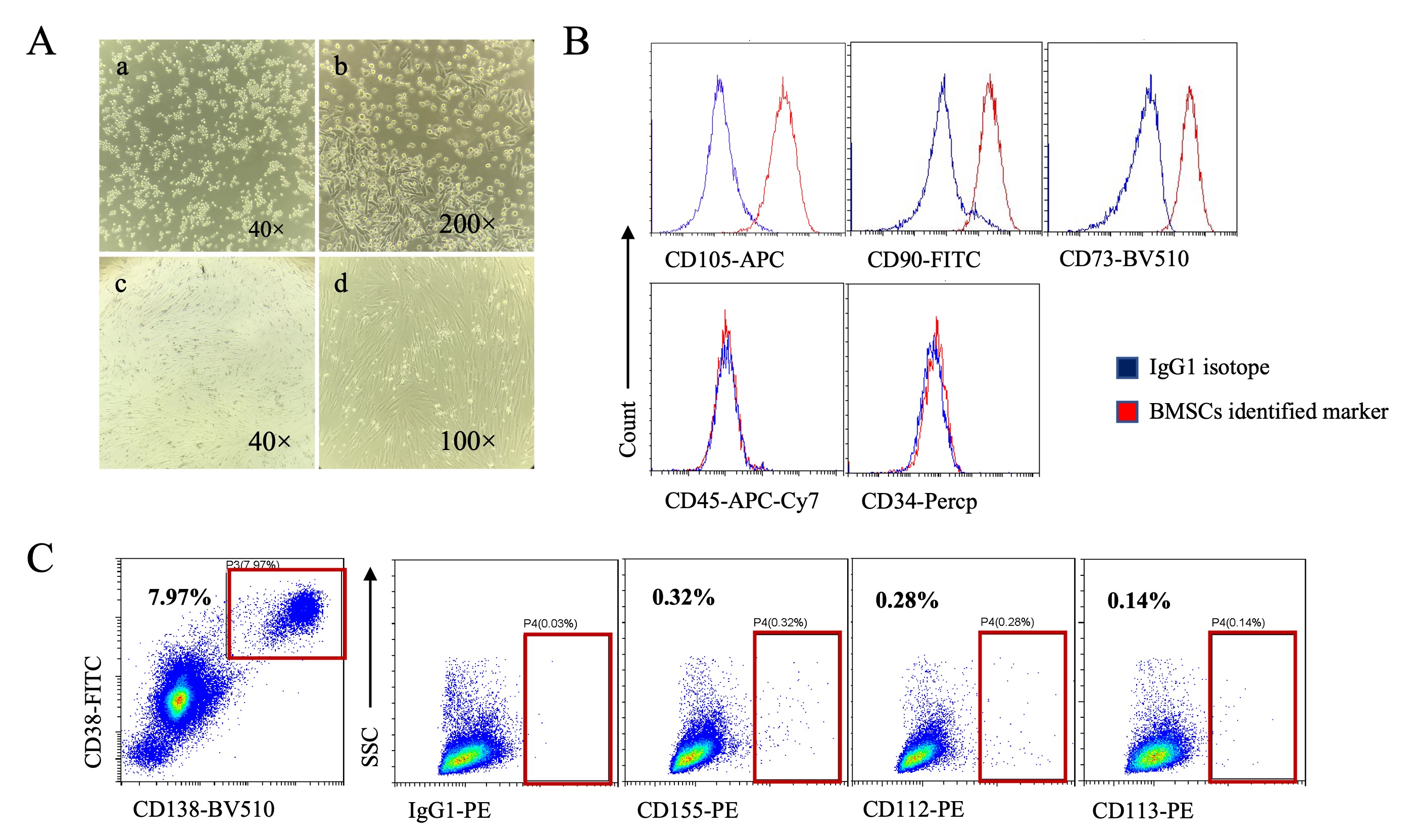

Supplement: Supplementary file 3 — Supporting Information [file CTM2-12-e861-s001.jpg]

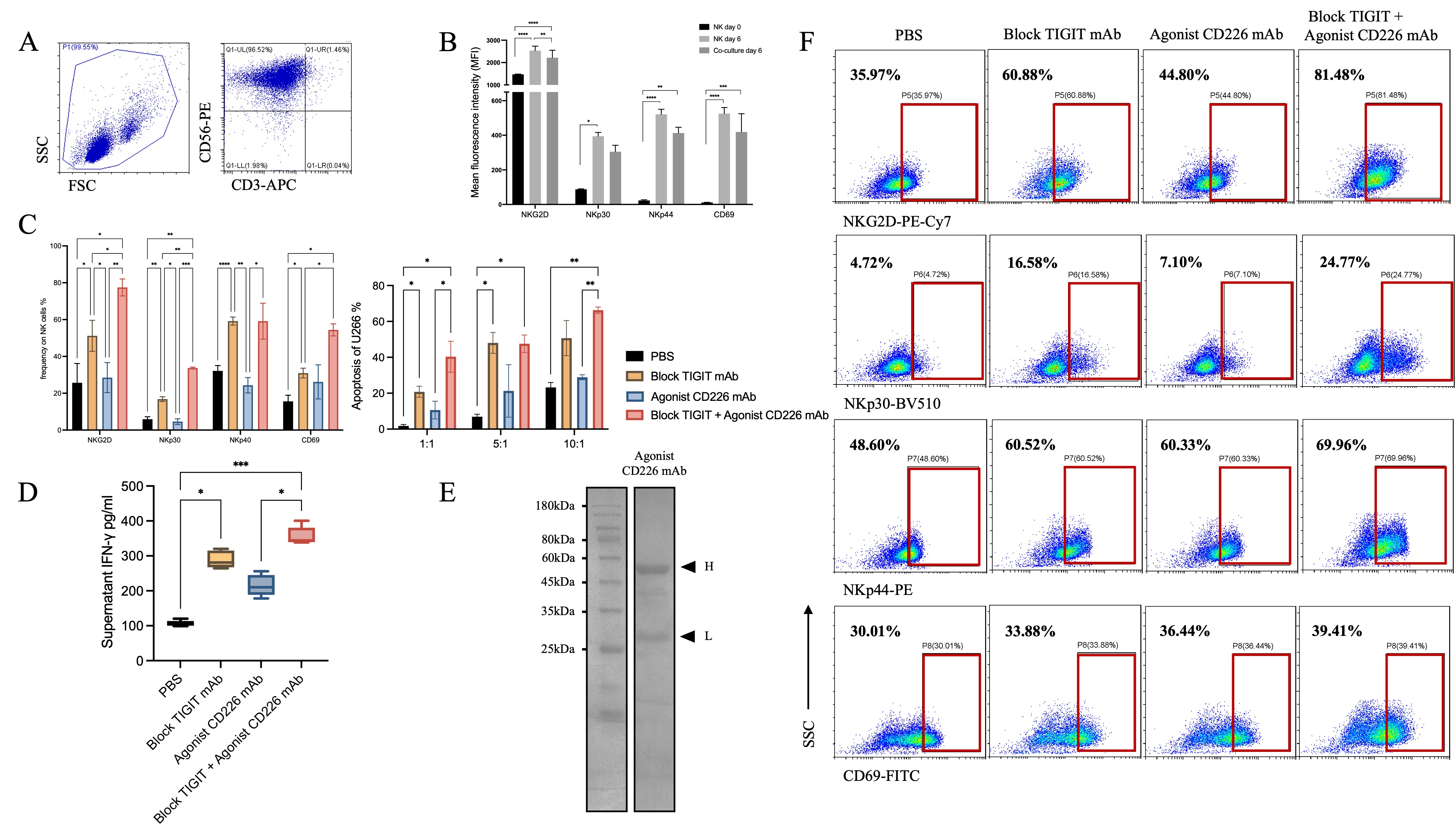

Supplement: Supplementary file 4 — Supporting Information [file CTM2-12-e861-s002.jpg]

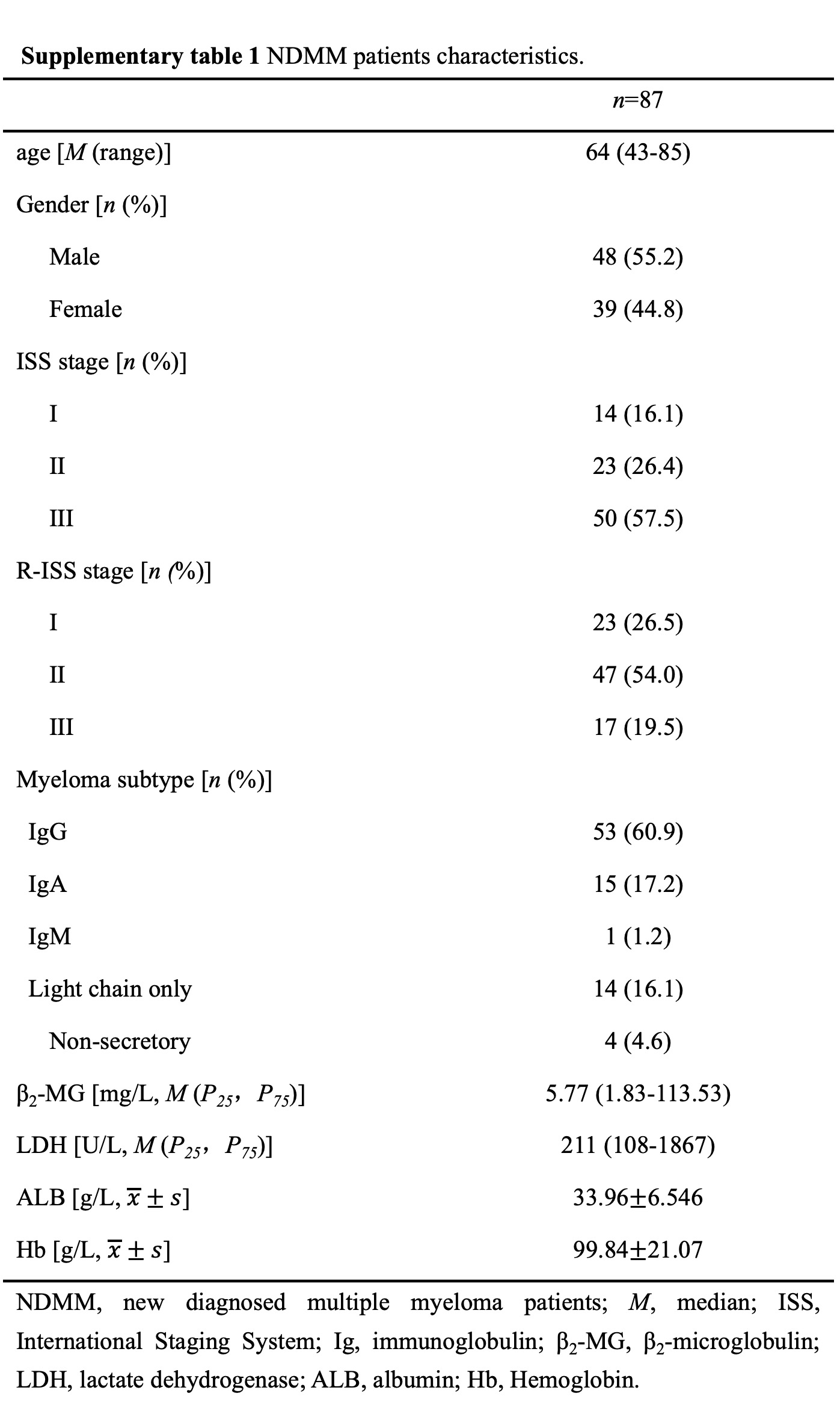

Supplement: Supplementary file 5 — Supporting Information [file CTM2-12-e861-s005.jpg]

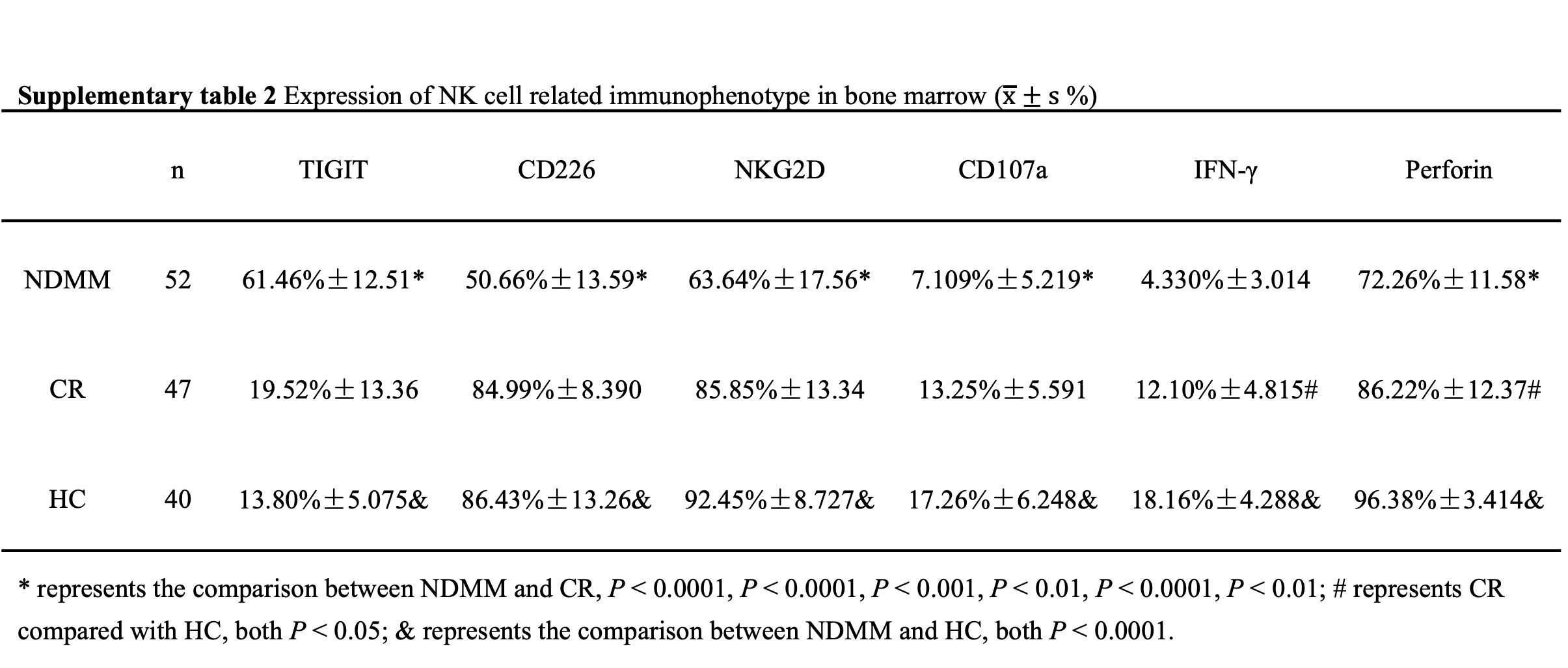

Supplement: Supplementary file 6 — Supporting Information [file CTM2-12-e861-s006.jpg]

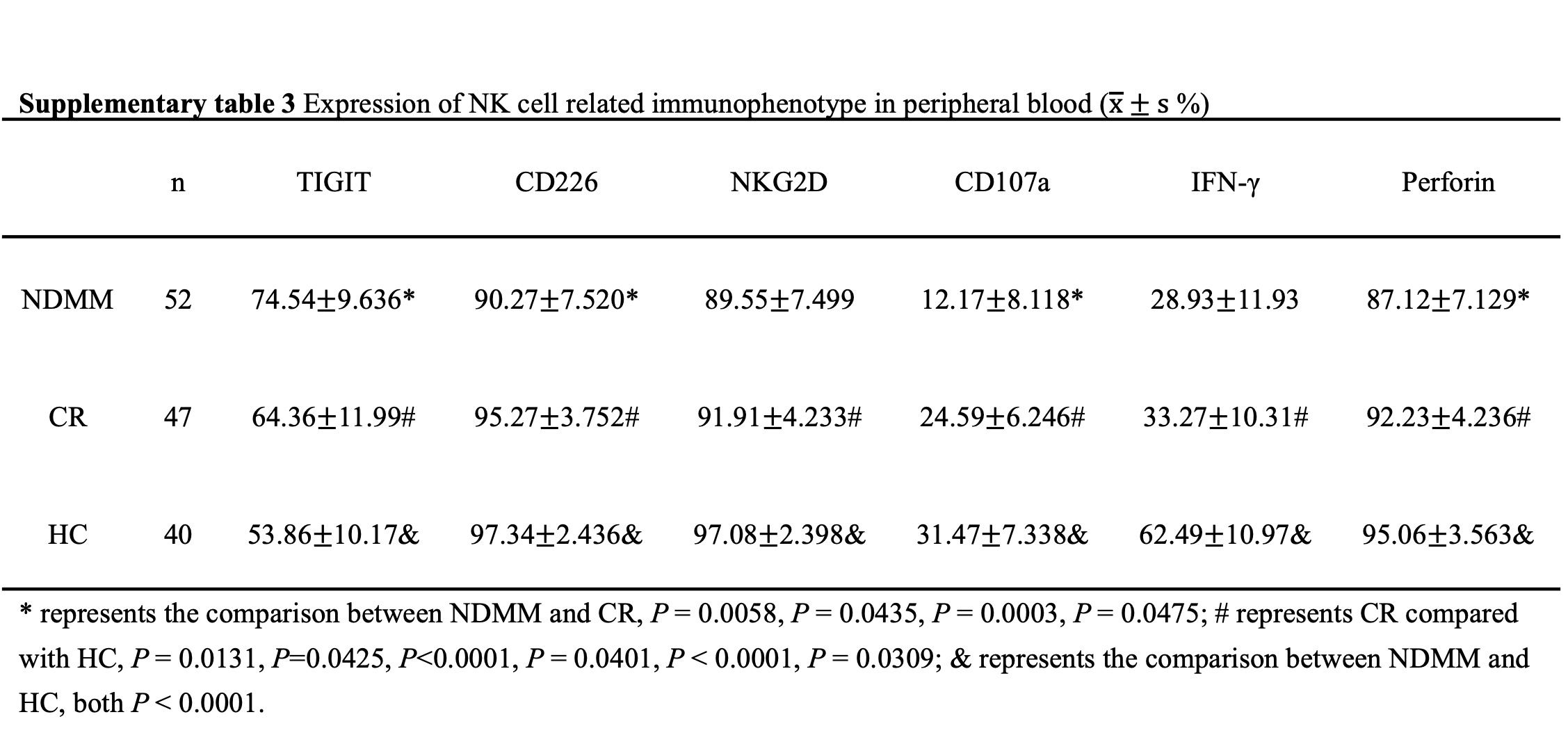

Supplement: Supplementary file 7 — Supporting Information [file CTM2-12-e861-s007.jpg]
